# A Retrospective Cohort Study of Total Colonic Aganglionosis: Is the Appendix a Reliable Diagnostic Tool?

**DOI:** 10.21699/jns.v6i1.514

**Published:** 2017-01-01

**Authors:** Carlos Albert Reck-Burneo, Victoria Lane, Prasad Vinay, Richard Wood, Marc Levitt

**Affiliations:** 1 Center for Colorectal and Pelvic Reconstruction, Nationwide Children’s Hospital, Columbus, Ohio, USA; 2 Leeds Children’s Hospital, Great George Street, West Yorkshire, LS1 3EX, UK; 3 Department of Pathology, Nationwide Children’s Hospital, Columbus, Ohio, USA

**Dear Sir**

O’Hare et al “A Retrospective Cohort Study of Total Colonic Aganglionosis: Is the Appendix a Reliable Diagnostic Tool?”[1] suggest that aganglionosis of the appendix is a reliable tool in the diagnosis of total colonic Hirschsprung’s disease. They base their assumption on a case series of 9 patients with total colonic aganglionosis and the finding of ab-sence of ganglion cells in the appendix.

We would like the authors to note the recent review on the subject in Lane et al. “The Appendix and Aganglionosis. A Note of Caution-How the Histology Can Mislead the Surgeon in Total Colonic Hirschsprung’s Disease”, [2] This report showed that the appendix is not reliable in determining whether there is Hirschsprung’s disease as many normal appendices have no ganglion cells. When we looked at this question in the reverse for a group of recent cases we found that in our last 4 cases of total colonic aganglionosis there were 3 cases in which ganglion cells were found in the appendix.

We believe the readership should recognize that using the appendix alone can mislead surgeons and we strongly caution against using it as a screening tool. We agree with the authors that further studies are needed and a better pathological understanding of the appendix is required. Factors including enterocolitis, inflammation and age can all affect the pathological finding complicating this issue further.

## Footnotes

**Source of Support:** Nil

**Conflict of Interest:** None
